# Anti-ultraviolet, antibacterial, and biofilm eradication activities against *Cutibacterium acnes* of melanins and melanin derivatives from *Daedaleopsis tricolor* and *Fomes fomentarius*

**DOI:** 10.3389/fmicb.2023.1305778

**Published:** 2024-01-08

**Authors:** Tu N. Le, Ngan T. H. Tran, Vy N. T. Pham, Ngoc-Dung Van-Thi, Hanh T. M. Tran

**Affiliations:** ^1^Applied Microbiology Laboratory, School of Biotechnology, International University – VNU HCM, Linh Trung ward, Thu Duc, Ho Chi Minh City, Vietnam; ^2^Research Group Experimental Pharmacology (EFAR), Department of Pharmaceutical Chemistry, Drug Analysis and Drug Information (FASC), Center for Neurosciences (C4N), Vrije Universiteit Brussel (VUB), Laarbeeklaan, Brussels, Belgium

**Keywords:** acne-causing bacterium, arginine-modified melanin, biofilm eradication, *Cutibacterium acnes*, fungal melanin, natural antibiotics

## Abstract

*Fomes fomentarius* and *Daedaleopsis tricolor* produced significant amounts of water-insoluble melanins, and our previous study successfully enhanced their water solubility by arginine modification. This research aimed to investigate the anti-ultraviolet, antibacterial, and biofilm eradication activities of both the melanins and arginine-modified melanin (melanin derivatives) from these two fungi against an acne-causing bacterium (*Cutibacterium acnes*). Apart from these, the cytotoxicity of the melanins and melanin derivatives on human skin cells was also evaluated. Melanin derivatives of both two fungi showed significantly higher antibacterial and biofilm eradication activities compared with their original forms. Specifically, the MIC_50_ values of the melanin derivatives (1,000 μg/mL) are the same as those of erythromycin. Regarding biofilm eradication capacity, the MBEC_50_ value of *D. tricolor* melanin derivative (250 μg/mL) was just half of both erythromycin and *F. fomentarius* melanin derivative. However, it required a 2-fold higher concentration of melanin derivatives than erythromycin to inhibit 90% of the bacterial population and eradicate 90% of their biofilm. Regarding anti-ultraviolet activity, blending melanins or melanin derivatives with a moisturizer/sunscreen enhanced their UV light absorption and the sun protection factor (SPF) values. In addition, melanins showed better effects than their derivatives, and those of *D. tricolor* were better than *F. fomentarius*. Remarkably, adding *D. tricolor* melanin (10%) to a Nivea pure cream could turn this cream into a broad-spectrum sunscreen, with its SPF value and critical wavelength increasing from 7.74 and 338.67 to 14.02 and 377.0, respectively. In addition, adding melanin or a melanin derivative of *D. tricolor* to an Olay sunscreen enhanced the SPF and the critical wavelength of the sunscreen from 17.25 and 371.67 to 23.82 and 374 and 23.38 and 372, respectively. Notably, melanins and melanin derivatives showed no toxicity in human fibroblasts. The obtained data suggest that arginine modification significantly enhanced the antibacterial and biofilm eradication activities of melanins from *D. tricolor* and *F. fomentarius*. However, this is not the case when it comes to their anti-ultraviolet activities. In addition, melanin and melanin derivatives from *D. tricolor* are potential candidates for anti-acne sunscreen products and are worth further investigation.

## Introduction

Melanins are black or brown pigments of high molecular weight formed by the oxidative polymerization of phenolic or indolic compounds (Pralea et al., 2019). Melanins are present in all kingdoms of life, from microorganisms (e.g., fungi) to higher organisms (e.g., humans), and they protect the hosts from harsh conditions (Li et al., 2018). Melanins have a wide spectrum of biological properties, including antimicrobial, anti-radiation, photoprotection, antioxidant, anti-aging, and anticancer activities (Pombeiro-Sponchiado et al., 2017; Suwannarach et al., 2019; El-Naggar, 2021).

In terms of antimicrobial activity, melanins from fungi and Streptomyces are well known for their profound antibacterial activities. For example, *Schizophyllum commune* melanin displayed significant growth inhibitory effects on *Escherichia coli*, *Proteus* sp., *Klebsiella pneumonia*, and *Pseudomonas fluorescens* (Arun et al., 2015). Similarly, melanin extracted from *Hortaea werneckii* also significantly inhibited *Lactobacillus vulgaris*, *E. coli*, and *Vibrio cholera* (Helan et al., 2013). In addition, *Armillaria mellea* melanins could inhibit the growth of *Bacillus cereus*, *B. subtilis*, *Enterococcus faecalis*, and *Pseudomonas aeruginosa* and melanins from *Exidia nigricans* and *Scleroderma citrinum* could inhibit the two latter species (Łopusiewicz, 2017, 2018a,b). More recently, Polapally et al. (2022) reported that *Streptomyces puniceus* melanin inhibited *Klebsiella pneumoniae* and *B. cereus*. The initial data suggested that melanins (from *Lachnum* sp.) inhibit/kill pathogenic bacteria (e.g., *Vibrio parahaemolyticus* and *Staphylococcus aureus*) by damaging their membranes (Xu et al., 2017).

*Cutibacterium acnes* is an opportunistic Gram-positive anaerobic bacterium that greatly contributes to acne vulgaris, which is one of the most prevalent skin conditions (McLaughlin et al., 2019). Although it is not a fatal disease, those with acne are prone to developing physiological distress and the risk of obstinate skin scarring. Several antibiotics could be used for acne treatment, including tetracyclines, erythromycin, metronidazole, nadifloxacin, clindamycin, and dapsone (Süer and Güvenir, 2019). However, due to the capability of forming biofilm, *C. acnes* has developed some levels of resistance to these antibiotics (Coenye et al., 2007; Sardana et al., 2015). Thus, searching for alternative antimicrobial compounds from natural materials is crucial. Previous studies found that essential oils from some plants, e.g., cloves, cacti, and tea trees, significantly inhibited the growth of *C. acnes* (Murbach Teles Andrade et al., 2018; Ossa-Tabares et al., 2020). There has not been any research attempting to investigate the antibacterial and antibiofilm activities of melanins against *C. acnes*.

Apart from their antimicrobial activities, melanins have polymeric structures with aromatic rings that make them naturally effective screeners against UVB and UVA radiation (Cockell and Knowland, 1999). Melanogenesis in human skin could absorb 50–75% of UV radiation and have a sun protection factor (SPF) of 1.5–2.0 (Brenner and Hearing, 2007). Anti-ultraviolet activities are also reported in fungal melanins. For example, *Agaricus bisporus* melanin effectively reduced light transmission at 315 nm. Remarkably, this melanin also significantly enhanced human skin cell viability when the cells were irradiated with a high dose of ultraviolet B (Olaizola et al., 2013). Blending the melanin of *Amorphotheca resinae* with a pure cream could enhance 2.5-fold the *in vitro* SPF value and broaden the protective spectra with a critical wavelength of approximately 388 nm (Oh et al., 2021).

Despite having remarkable bioactivities, most melanins are only soluble in alkaline solutions, but insoluble in water and other solvents; thus, it is challenging to bring them to applications (Pralea et al., 2019). Fortunately, initial studies found that melanin modification with amino acids could enhance their water solubility and subsequently, some of their bioactivities. For example, modification of fungal melanin with either arginine or glucosamine increased the anti-radiation, antioxidant, and antitumor activities of the samples (Ye et al., 2012; Song et al., 2016; Li et al., 2018; Shi et al., 2018; Xu et al., 2020).

*Daedaleopsis tricolor* and *Fomes fomentarius* are medicinal mushrooms that form large numbers of (large) fruiting bodies in forests. Our previous research successfully extracted and purified melanins from the fruiting bodies of these fungi and modified their melanins with arginine (the attachment of arginine molecules to the melanins was confirmed by FTIR analysis). The water solubility of the arginine-modified melanins (melanin derivatives) was found to be significantly enhanced. Therefore, the objectives of this research were to evaluate the antibacterial and biofilm eradication activities against *C. acnes* of melanins and melanin derivatives from *D. tricolor* and *F. fomentarius*. A part of the research also investigated their anti-ultraviolet properties and cytotoxicity (if any) toward human skin cells.

## Materials and methods

### Microbial samples

*Daedaleopsis tricolor* and *F. fomentarius* were collected from Brussels (Belgium) in December 2022 and molecularly identified using ITS sequencing (Supplementary File 1).

*Cutibacterium acnes* was kindly given by Dr. Bich from Tay Do University (Vietnam). The bacterium was cultured in brain heart infusion (BHI) and incubated at 37°C under anaerobic conditions.

### Melanin extraction and purification

Melanin was extracted and purified following Rajagopal et al. (2011) and Elsayis et al. (2022) with some modifications. The fungal fruiting body powder was mixed with 1 M KOH solution (1:40, w/v), incubated for 48 h at room temperature, then centrifuged at 8,000 rpm for 15 min, and the supernatant was collected. The pH of the collected supernatant was adjusted to 2.5 using 2 M HCl, and the mixture was incubated at room temperature for 2 h for melanin precipitation. The pellet was then collected after centrifugation and washed with distilled water three times to remove the excess acid before being freeze-dried.

The purification was conducted by boiling the crude melanin in 7 M HCl at 100°C for 2 h, followed by centrifugation at 10,000 rpm for 20 min. After that, the collected pellet as purified melanin was washed with 0.01 M HCl three times and then freeze-dried for further use (El Bassam et al., 2002; Rajagopal et al., 2011; Elsayis et al., 2022).

### Melanin modification

The purified melanin samples were modified with L-arginine following Xu et al. (2020) with some modifications. Based on our previous study, the arginine was mixed with *F. fomentarius* melanin in a ratio of 1:1 (w/w) and 1:1.5 (w/w) with *D. tricolor* melanin, and then dissolved in 5 mL of distilled water. The mixture was then incubated at 37°C for 45 min before being centrifuged at 8000 rpm for 10 min. The supernatant was collected, freeze-dried, and stored at 4°C for further use (Xu et al., 2020).

### Antibacterial assay

The 3-day-old *C. acnes* bacterial suspension was standardized to 5 × 10^7^. Melanin samples were dissolved in 5% DMSO to achieve a concentration range of 31.25 to 2000 μg/mL, and the modified melanins were dissolved in sterile water to achieve a concentration range of 62.5 to 8,000 μg/mL. It should be noted that due to the low solubility of melanin in water as well as 5% DMSO, the actual concentrations of melanin in 5% DMSO were lower than the target concentrations mentioned here (31.25 to 2,000 μg/mL). The undissolved melanin was removed from the solution, but the concentrations (31.25 to 2,000 μg/mL) were still labeled based on the amount of melanin added to a known volume of 5% DMSO for comparison purposes.

A volume of 100 μL of bacterial suspension was dispersed into each well of a sterile 96-well plate, followed by the addition of 100 μL of either melanin or modified melanin. The negative control well consisted of 100 μL of bacterial suspension and either 100 μL of DMSO 5% or 100 μL of distilled water, whereas the positive control consisted of 100 μL of erythromycin solution and 100 μL bacterial suspension. The optical density at 600 nm of the plate was measured by a Cytation 3 multimode plate reader (BioTek, Winooski), and the percentage of growth inhibition was determined using the following formula (Quave et al., 2008; Nelson et al., 2016):


$$\%\mathrm{inhibition}=\left(1-\frac{OD_{72h}-OD_{0h}}{OD_{\left(-\right)\mathrm{control}}}\right)\times 100$$

where: OD_72h_ is the optical density (600 nm) of the test well post-inoculation; OD_0h_ is the optical density (600 nm) of the test well at 0 h post-inoculation; and OD_(−)control_ is the optical density (600 nm) of the negative control well post-inoculation.

The minimal concentration of the sample required to inhibit the growth of the bacterial population by 50% (MIC_50_) and 90% (MIC_90_) was calculated from the obtained MIC data.

### Minimum biofilm eradication concentration (MBEC) assay

The MBEC experiment was compared to the demonstrations of Nelson et al. (2016) and Pannu et al. (2011). A volume of 100 μL of standardized bacterial suspension was added into a sterile tissue culture round-bottom 96-well plate. The plate was then incubated anaerobically for 48 h for biofilm formation. The culture broth was then gently removed, and fresh medium with melanin or modified melanin was added. The final concentration in the wells of melanin was from 15.625 to 4,000 μg/mL, and that of modified melanin was from 31.25 to 8,000 μg/mL (the difference in the final concentration range was due to the difference in their solubility). The negative control wells consisted of 100 μL of bacterial suspension and either 100 μL of DMSO 1.2% or 100 μL of distilled water, whereas the positive control consisted of 100 μL of bacterial suspension and 100 μL of erythromycin in 1.2% DMSO/ distilled water. The formed biofilms were incubated with the sample for another 48 h; after that, the broth was removed. Biofilm staining was subsequently performed after the wells were rinsed with PBS three times and heat-fixed at 37°C for 1 h. The biofilms were then stained with 200 μL of 0.1% crystal violet for 15 min, and the excess stain was rinsed with distilled water. After being dried, the 96-well plate was treated with 200 μL of 10% Tween 80 (in EtOH) to elute the stain. Finally, 150 μL of the stain solution was transferred to a new 96-well plate for measuring the optical density at 595 nm.

The percentage of biofilm eradication was then calculated by the following equation:


$$\%\mathrm{biofilm}\;\mathrm{eradication}=\left(1-\frac{OD_{\mathrm{sample}}}{OD_{\left(-\right)\mathrm{control}}}\right)\times 100$$

where OD_sample_ is the absorbance value of each concentration of the melanin/melanin derivative; OD_blank_ is the absorbance value of the blank control (DMSO/distilled water); and OD_(−)control_ is the absorbance value of the negative control.

The minimum concentrations of studied samples that would eradicate 50% (MBEC_50_) and 90% (MBEC_90_) of the biofilm formation were calculated based on the biofilm eradication against the tested concentration of the sample (Nelson et al., 2016).

### Evaluating SPF values of melanin-blended creams

Nivea Soft Moisturizing Cream (no label of sun protection factor) and Olay Total Effects Moisturizer (SPF 15) were used as cream bases for the melanin-blended cream formulation. Cetaphil Daily Facial Moisturizer (SPF 15) was a reference cream to validate the accuracy of the method. Different melanin/derivate concentrations (4, 7, and 10%, w/w) were mixed with the Nivea Soft Moisturizing Cream or Olay Total Effects Moisturizer (SPF 15). 0.1 M NaOH or distilled water was used as the solvent. The detailed ingredients used for different formulas of blended cream are depicted in Supplementary Table S1.

To measure the SPF index of the melanin-blended cream, the Mansur et al. (1986) method was applied with some modifications. A volume of 20 mL of absolute ethanol was added to dissolve the blended cream sample, followed by 5-min–130 W and 20 kHz ultrasonication. After that, 2 mL of the solution was transferred to a clean container and filled up to 10 mL with absolute ethanol. The sample was then transferred to a 1-cm quartz cuvette. The absorbance spectra of the mixture were scanned by a UV visible spectrophotometer within a range of 290–400 nm, every 1 nm. Consequently, sun protection factor (SPF) values were determined using the equation proposed by Mansur et al. (1986), as follows:


$$SPF_{\mathrm{spectrophotometric}}=CF\times \overset{320}{\underset{290}{\sum }}EE\left(\lambda \right)\times I\left(\lambda \right)\times \mathrm{Abs}\left(\lambda \right)$$

where EE (λ) is the erythemal effect spectrum; I (λ) stands for solar intensity spectrum; Abs (λ) represents the absorbance of lotion/ sunscreen product/melanin-blended cream; and *CF* indicates the correction factor, which equals 10. The values of EE x I are constants with the corresponding wavelength, adopted from Sayre et al. (1979).

For testing the broad-spectrum protection characteristic of melanin, the following index, which was given by Diffey (1994) was applied.


$$R=\overset{\lambda }{\underset{290}{\int }}A_{\lambda }d\lambda ∕\overset{400}{\underset{290}{\int }}A_{\lambda }d\lambda$$

where A_λ_ stands for spectral absorbance value, and d_λ_ illustrates the wavelength interval between measurements. The critical wavelength is defined as the wavelength at which the R ≥ 0.9, corresponding to 90% and above of the total area under the UV absorbance curve. Specifically, a product that reaches 370 nm or longer could be claimed as a broad-spectrum sunscreen.

### Evaluating cytotoxicity of melanins and melanin derivatives on human fibroblast cells

The human normal fibroblast cell line used in this research was established from an adult fresh foreskin sample (Nguyen and Ho-Huynh, 2016). The cells were cultured in DMEM/F12 containing 10% (v/v) FBS, 20 mM HEPES, 0.025 g/mL of amphotericin B, 100 IU/mL of penicillin G, and 100 g/mL of streptomycin at 37°C and 5% CO_2_.

The cytotoxicity of the fungal melanin and melanin derivative samples on fibroblasts was determined by sulforhodamine B (SRB) assay, as previously described by Nguyen and Ho-Huynh (2016). The fibroblast cells were grown on 96-well plates for 24 h at a density of 10^4^ cells/well before being treated with the melanin/derivative sample at the desired concentration for 48 h. The cells were fixed for 1–3 h with a cold 50% (w/v) trichloroacetic acid (Merck) solution, washed, and stained for another 20 min with 0.2% (w/v) sulforhodamine B (Sigma). The plates were washed five times with 1% acetic acid (Merck) to remove unbound dye. Then, the protein-bound dye was solubilized in 10 mM Tris base solution (Promega). At 492 nm and 620 nm wavelengths, optical density values were obtained using a 96-well microtiter plate reader (Synergy HT, Biotek Instruments). The positive control being used was camptothecin (Calbiochem). The experiment was replicated three times.

The growth inhibition percentage was calculated using the following formula:


$$\%\mathrm{growth}\;\mathrm{inhibition}\;\mathrm{percentage}\;\left(Inh\%\right)=\left(1-\frac{OD_{t}}{OD_{c}}\times 100\right)\%$$

where OD_t_ and OD_c_ represent the optical density values of the tested and control samples, respectively (Nguyen and Ho-Huynh, 2016).

### Data analysis

All the experiments were conducted in triplicate, and ANOVA test was run (SPSS software, version 29) for comparisons of multi-group data. If the value of *p* was <0.05, the case was considered significantly different. The obtained data were displayed as mean ± standard deviation.

## Results

### Antibacterial activity of melanin and melanin derivatives against *Cutibacterium acnes*

**Figure 1:**
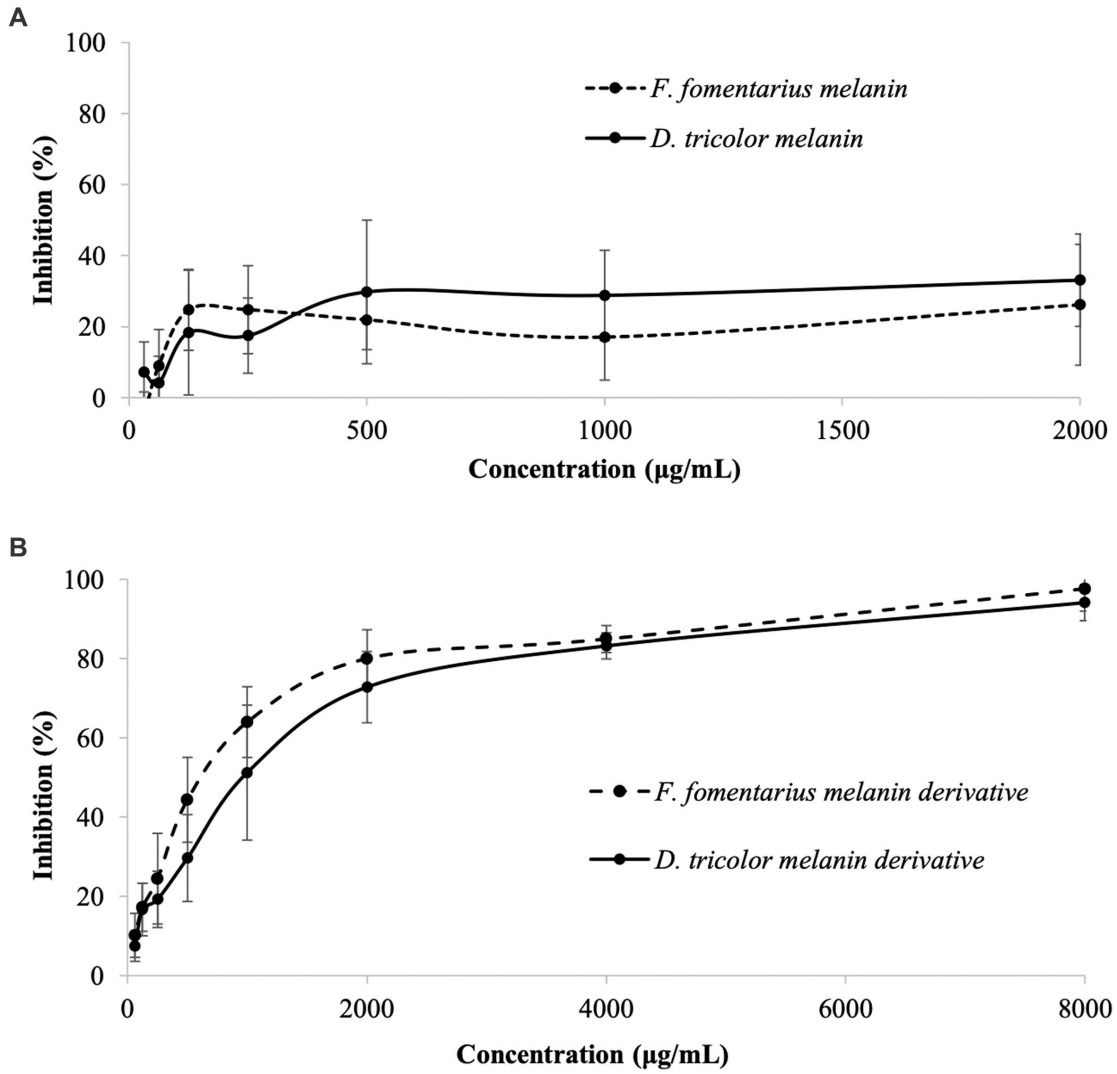
Antibacterial activities against *Cutibacterium acnes* of **(A)** *Daedaleopsis tricolor* and *Fomes fomentarius* melanins at concentrations of 31.25–4,000 (μg/mL) dissolved in DMSO and **(B)** melanin derivatives at concentrations of 62.5–8,000 (μg/mL) dissolved in sterile distilled water.

Antibacterial activities of different concentrations of melanins and melanin derivatives against *C. acnes* and their MBEC_50_ and MBEC_90_ values are displayed in Figure 1 and Table 1. As mentioned in the method section, due to the limited solubility in DMSO, the actual concentrations of melanins in the test wells were much lower than 31.25–4,000 μg/mL. After mixing melanin with DMSO, the undissolved amount of melanin was then removed from the solution, but the solution was still labeled based on the amount of melanin added to a known volume of 5.0% DMSO at the beginning for easy comparison with the melanin derivatives.

**Table 1:** MIC_50_ and MIC_90_ values of *Fomes fomentarius* and *Daedaleopsis tricolor* melanins and melanin derivatives against *Cutibacterium acnes.*

Sample	MIC_50_ (mg/mL)	MIC_90_ (mg/mL)
Erythromycin (positive control)	<1,000	>4,000
*D. tricolor* melanin	ND	ND
*F. fomentarius* melanin	ND	ND
*D. tricolor* melanin derivative	1,000	8,000
*F. fomentarius* melanin derivative	<1,000	8,000

ND, not detected.

Generally, both melanins and melanin derivatives displayed antibacterial activity against *C. acnes*. The antibacterial activities of the melanin derivatives were dose-dependent, and the activities significantly increased along with the increase in sample concentration (Figure 1B). However, this is not the case with melanins. Due to their limited solubility in DMSO and distilled water, the melanin samples displayed insignificant antibacterial activities, which were the same at all the tested concentrations (Figure 1A). Thus, it was impossible to detect their MIC_50_ and MIC_90_ values. Remarkably, the MIC_50_ values of the melanin derivatives (1,000 μg/mL) are the same as those of the positive control (erythromycin).

It should be noted that arginine with different concentrations was evaluated against *C. acnes*, and the data found that arginine alone had no antibacterial activity against this bacterium (Supplementary Table S2).

### Biofilm eradication activity of melanin and melanin derivatives against *Cutibacterium acnes*

**Figure 2:**
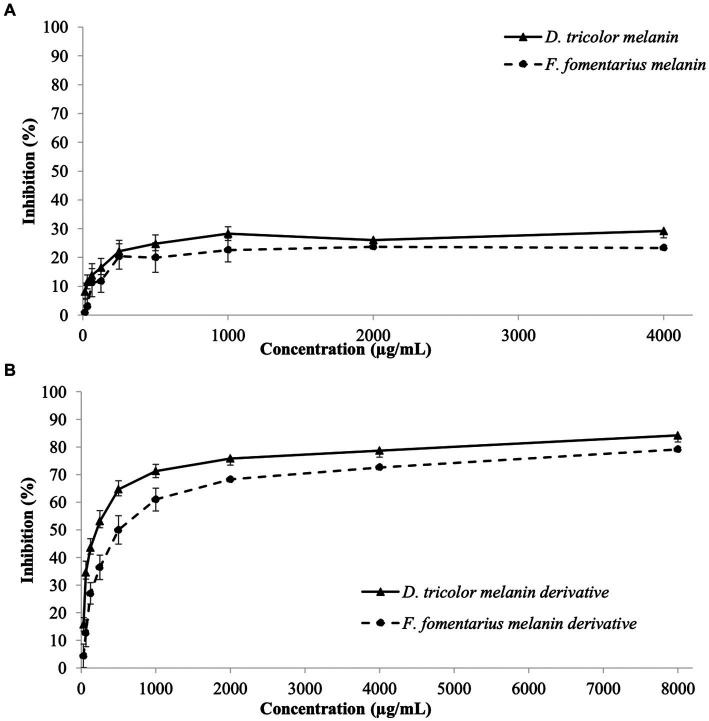
Biofilm eradication activities against *Cutibacterium acnes* of **(A)** *D. tricolor* and *F. fomentarius* melanins at concentrations of 31.25–4,000 μg/mL dissolved in DMSO and **(B)** melanin derivatives at concentrations of 62.5–8,000 (μg/mL) dissolved in sterile distilled water.

Antibiofilm activities of the tested samples against *C. acnes* are displayed in Figure 2. Their MBEC_50_ and MBEC_90_ values are presented in Table 2.

**Table 2:** MBEC_50_ and MBEC_90_ values of *F. fomentarius* and *D. tricolor* melanins and melanin derivatives against *C. acnes.*

Sample	MBEC_50_ (μg/mL)	MBEC_90_ (μg/mL)
Erythromycin (control)	500	4,000
*D. tricolor* melanin	ND	ND
*F. fomentarius* melanin	ND	ND
*D. tricolor* melanin derivative	250	>8,000
*F. fomentarius* melanin derivative	500	>8,000

ND, not detected.

Both *D. tricolor* and *F. fomentarius* melanin derivatives exhibited dose-dependent effects against *C. acnes* biofilm formation (Figure 2B). In terms of MBEC_50_, the *D. tricolor* melanin derivative (250 μg/mL) displayed significantly higher activity than that of the *F. fomentarius* melanin derivative (500 μg/mL) and the positive control (500 μg/mL). However, both *D. tricolor* and *F. fomentarius* derivatives might require a concentration greater than 8,000 μg/mL to inhibit 90% of *C. acnes* biofilm formation, whereas that of the positive control was 4,000 μg/mL.

Like the case of the antibacterial activity described above, the melanin samples displayed insignificant antibiofilm activity, which was indifferent at all tested concentrations. Therefore, it was impossible to detect their MBEC_50_ and MBEC_90_ values.

To confirm that the antibiofilm activity was of the arginine modified melanins and was not because of the unbound arginine present in the melanin derivatives, antibiofilm activity of arginine was tested using the same method. The obtained data showed that arginine had no antibiofilm activity at all the tested concentrations (31.25–8,000 μg/mL) (Supplementary Table S3).

### Anti-ultraviolet activity and SPF of the melanin-blended creams

The efficacy of sunscreen can be depicted via its sun protection factor (SPF), which indicates the shielding ability of the sunscreen from UVB. Higher SPF values mean better protection of the product against sunburn.

**Figure 3:**
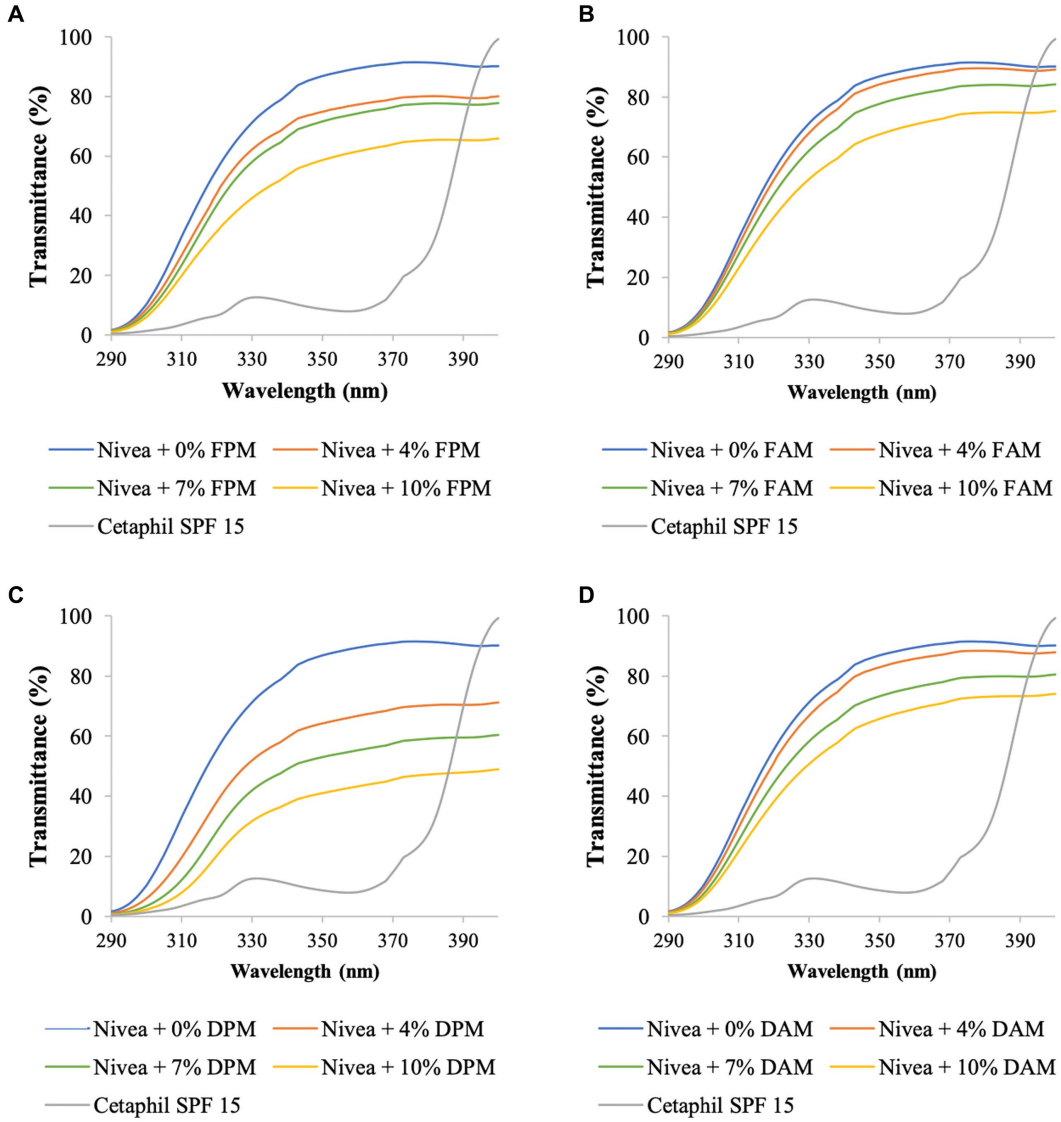
UV transmittance of Nivea cream blended with different concentrations of **(A)** *F. fomentarius* melanin (FPM), **(B)** *F. fomentarius* melanin derivative (FAM), **(C)** *D. tricolor* melanin (DPM), **(D)** *D. tricolor* melanin derivative (DAM). Cetaphil sunscreen (SPF 15) serves as the reference.

**Figure 4:**
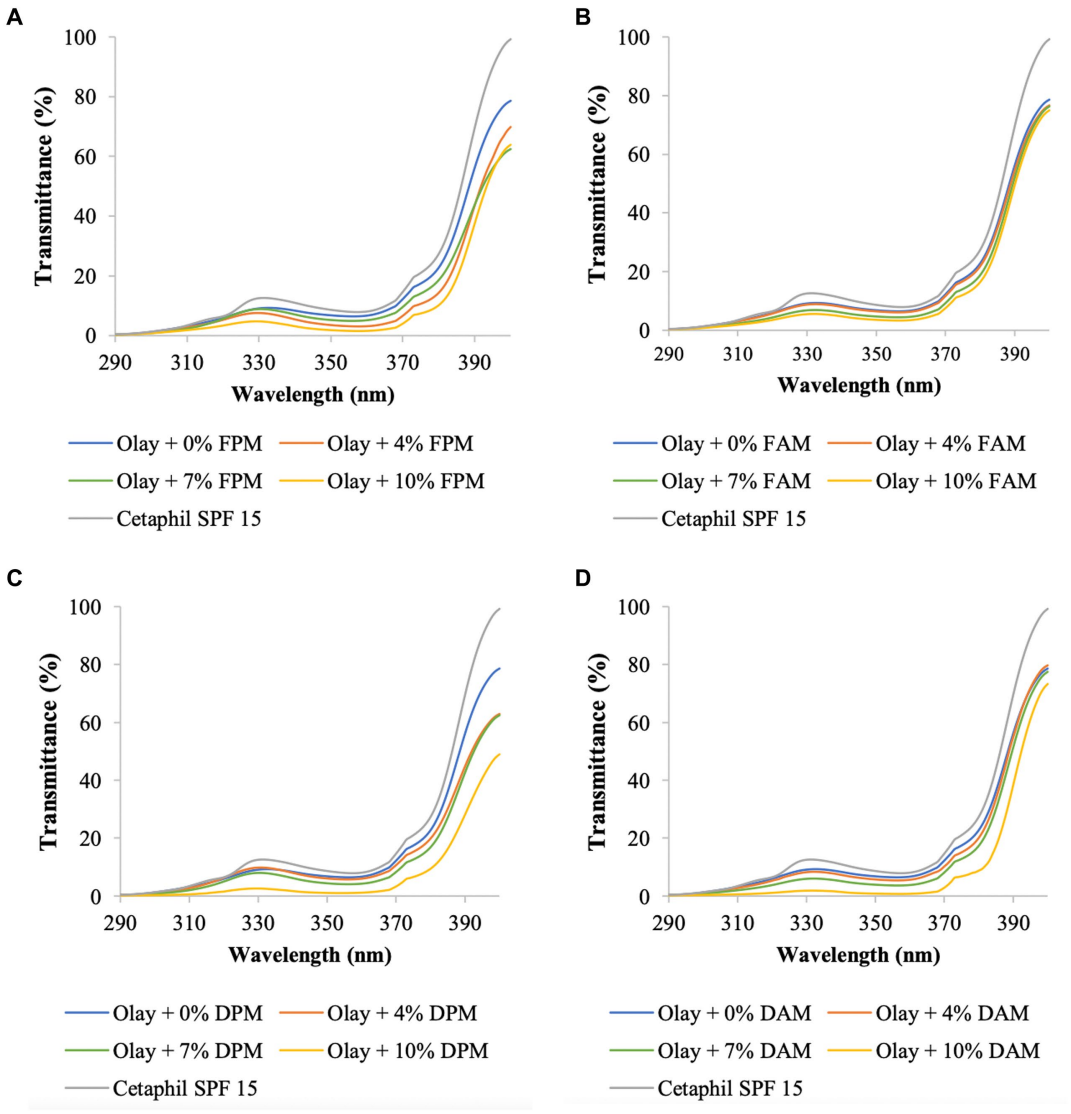
UV transmittance of Olay sunscreen blended with different concentrations of **(A)** *F. fomentarius* melanin (FPM), **(B)** *F. fomentarius* melanin derivative (FAM), **(C)** *D. tricolor* melanin (DPM), **(D)** *D. tricolor* melanin derivative (DAM). Cetaphil sunscreen (SPF 15) serves as the reference.

In this part of the study, melanin or melanin derivatives were blended with Nivea cream or Olay sunscreen with different concentrations to make blended creams. UV transmittance and SPF values of the melanin/melanin derivative-blended creams, positive control, and negative control are presented in Figures 3, 4, and Tables 3, 4.

**Table 3:** Sunscreen index of Nivea Soft Moisturizing Cream blended with different amounts of *F. fomentarius* and *D. tricolor* melanins and melanin derivatives.

Sample	Melanin sample	Melanin content	SPF	Critical wavelength
Nivea (Blank)	None	0% melanin	7.74 ± 0.2903^b^	338.67 ± 0.5
Cetaphil (+ control)	None	0% melanin	17.00 ± 0.1708^a^	370 ± 0
*F. fomentarius*	Melanin	4% melanin	8.53 ± 0.1847^b^	361.67 ± 0.5
		7% melanin	9.17 ± 0.1529^c^	363.33 ± 0.9
		10% melanin	9.99 ± 0.0179^d^	373 ± 0
	Melanin derivative	4% melanin	8.05 ± 0.0205^b^	345.67 ± 0.9
		7% melanin	8.48 ± 0.0348^b^	360.33 ± 0.5
		10% melanin	9.31 ± 0.0437^c^	366.67 ± 0.5
*D. tricolor*	Melanin	4% melanin	9.99 ± 0.2027^d^	369 ± 0.8
		7% melanin	12.21 ± 0.0209^e^	373.67 ± 0.5
		10% melanin	14.02 ± 0.0214^f^	377 ± 0
	Melanin derivative	4% melanin	8.22 ± 0.1619^b^	342.67 ± 0.5
		7% melanin	8.99 ± 0.1198^c^	354.67 ± 0.5
		10% melanin	9.68 ± 0.1775^d^	365.67 ± 0.5

Different superscript letters “a–f” exhibit the significant difference among recorded SPF values the samples.

**Table 4:** Sunscreen index of Olay Total Effect Sunscreen (SPF 15) blended with different concentrations of *F. fomentarius* and *D. tricolor* melanins and melanin derivatives.

Sample	Melanin sample	Melanin content	SPF	Critical wavelength
Olay (Blank)	None	0% melanin	17.25 ± 0.0125^a^	371.67 ± 0.5
Cetaphil (control)	None	0% melanin	17.00 ± 0.1708^a^	370 ± 0
*F. fomentarius*	Melanin	4% melanin	17.82 ± 0.2688^b^	374 ± 0
		7% melanin	18.66 ± 0.1656^c^	373.67 ± 0.5
		10% melanin	20.17 ± 0.1531^e^	374 ± 0
	Melanin derivative	4% melanin	17.47 ± 0.0968^a^	372 ± 0
		7% melanin	18.56 ± 0.1256^c^	371.67 ± 0.5
		10% melanin	19.34 ± 0.1323^d^	373 ± 0
*D. tricolor*	Melanin	4% melanin	17.99 ± 0.1126^b^	374 ± 0
		7% melanin	19.16 ± 0.1219^d^	374 ± 0
		10% melanin	23.82 ± 0.2340^f^	374 ± 0
	Melanin derivative	4% melanin	17.81 ± 0.1274^b^	372 ± 0
		7% melanin	19.11 ± 0.1577^d^	372 ± 0
		10% melanin	23.38 ± 0.1122^f^	372 ± 0

Different superscript letters “a–f” exhibit the significant difference among recorded SPF values the samples.

To validate the accuracy of the method, the data on SPF and UV transmittance labeled on the Cetaphil sunscreen and the Olay sunscreen were compared with the data obtained from our analysis. The measured SPF of Cetaphil sunscreen was around 17.0 and that of Olay sunscreen was 17.2, while both their SPF values are labeled as 15. Therefore, it would be safe to conclude that the protocol used in this research is reliable.

The UV transmittance of the blended creams significantly decreased (Figures 3, 4), and their SPF values significantly increased when increasing the concentrations of melanins or melanin derivatives from 4 to 10% (Tables 3, 4). These indicated that these melanin and melanin derivative samples could shield from UV rays and enhance the SPF of sunscreen products. This is especially obvious for the *D. tricolor* melanin-blended creams since the UV light absorption and SPF values are significantly different among the tested concentrations (4–10%) (*p* < 0.05). Evidently, melanin from *D. tricolor* was more effective than that from *F. fomentarius* (Tables 3, 4). In addition, melanin samples displayed significantly higher anti-ultraviolet and SPF values compared with the modified melanins, especially when blended with Nivea cream. This might be explained by the presence of L-arginine (in the ariginine-modified melanins), which is documented to be moisture- and light-sensitive (ThermoFisher SCI, 2009). In addition, in this part of the study, NaOH was used as the solvent for the preparation of the blended melanin creams, and melanins dissolved well in NaOH.

Regarding melanin-blended Nivea cream, the best treatment was found to consist of 10% *D. tricolor* melanin. This blended cream increased the SPF and critical wavelength of Nivea cream from 7.74 and 338 to 14.02 and 377, respectively (Table 3). This blended cream is considered a broad-spectrum anti-UVA and UVB cream (as the critical wavelength is greater than 370).

Olay sunscreen itself already had the SPF and critical wavelength values of 17.25 and 371, respectively. Blending this sunscreen with 10% *D. tricolor* melanin or melanin derivative enhanced these values to 23.82 and 374 and 23.38 and 372, respectively.

### Cytotoxicity of melanin and melanin derivatives

**Figure 5:**
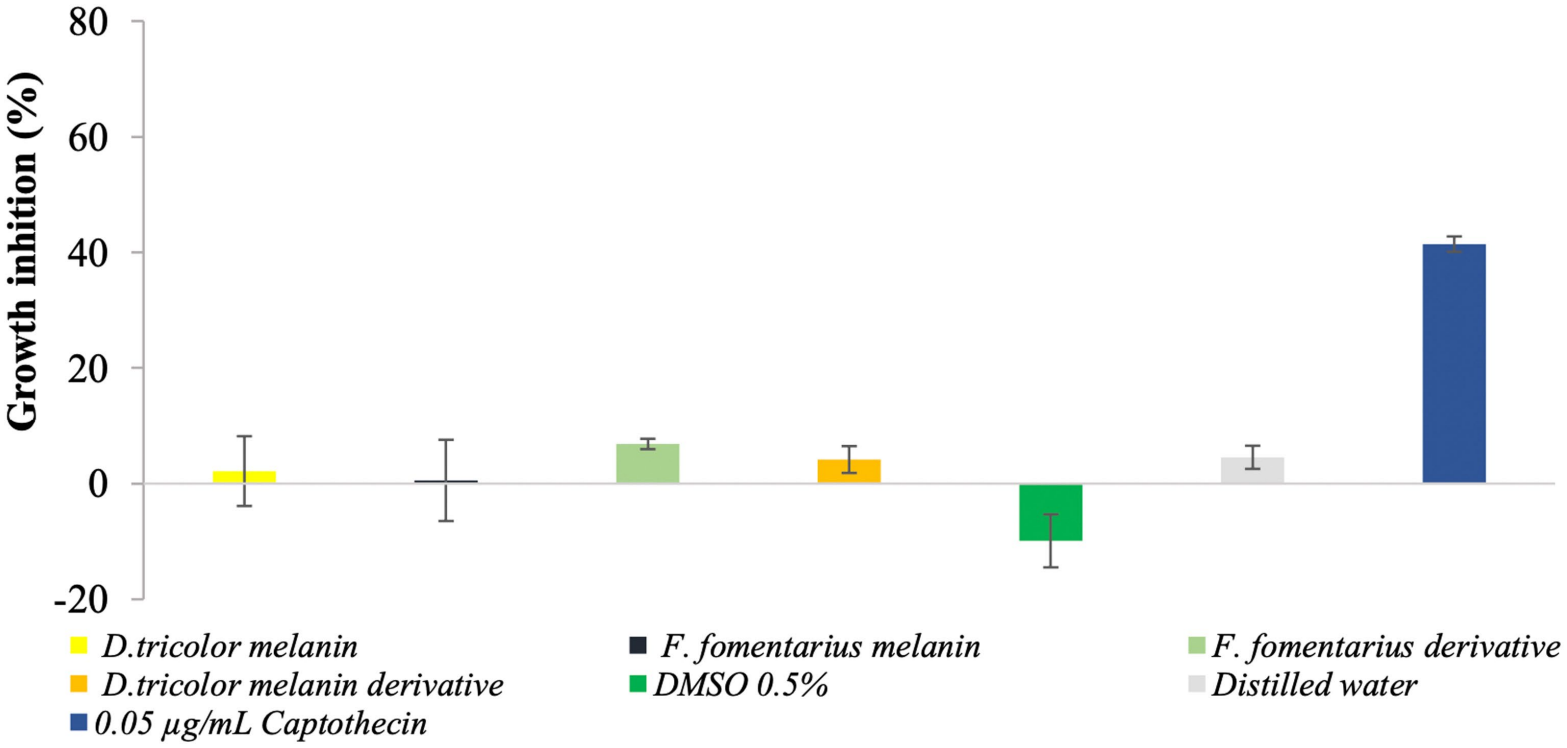
Cytotoxicity of *D. tricolor* and *F. fomentarius* melanins and melanin derivatives against fibroblast cells.

The cytotoxicity of purified fungal melanin and melanin derivatives (100 μg/mL) was assessed on normal fibroblasts using the sulforhodamine B (SRB) assay and Camptothecin as the positive control. The obtained data are shown in Figure 5.

Figure 5 shows that at a concentration of 100 μg/mL, the inhibition rates of melanins from *F. fomentarius* and *D. tricolor* against the skin cells were 0.55 and 2.16%, respectively. In addition, those of the melanin derivatives were 6.85 and 4.16%. These data were insignificant compared with the negative control (0.5% DMSO).

## Discussion

### Melanin derivatives from *Daedaleopsis tricolor* and *Fomes fomentarius* displayed significant antibacterial and biofilm eradication activities against *Cutibacterium acness*

Melanin derivatives of *D. tricolor* and *F. fomentarius* melanins, especially those of the former species, had comparable antibacterial activities with that of the positive control (Table 1). To the best of our knowledge, there has not been any report on the antibacterial activity of melanins against *C. acnes*. However, there have been several publications on the antibacterial activities of fungal melanins toward other pathogenic bacteria. For example, melanin from *Auricularia auricula* inhibited *E. coli*, *P. aeruginosa*, and *P. fluorescens* with MIC_50_ values of 160, 640, and 320 μg/mL, respectively (Bin et al., 2012). In another research, *Lachnum* YM30 intracellular melanins were found to inhibit different groups of bacteria, and their MBC values toward *Bacillus megaterium, Escherichia coli, Listeria monocytogenes, Salmonella typhi, Vibrio parahaemolyticus*, and *Staphylococcus aureus* were 0.4, 3.2, 0.3, 0.8, 0.8, and 0.4 mg/mL, respectively. Gram-negative bacteria are normally trickier to treat due to the presence of an additional membrane layer in their cell walls, which prevents antibiotics from penetrating deeper into the bacterial cells. However, based on these references, it seems fungal melanins are equally effective against both Gram-positive and Gram-negative bacteria, as well as bacteria with different oxygen requirements. Initially, Xu et al. (2017) reported that melanin from *Lachnum* YM30 damaged the integrity of the membranes and caused cell leaks in both Gram-negative bacteria (*Vibrio parahaemolyticus*) and Gram-positive bacteria (*Staphylococcus aureus*). The melanin derivatives from *D. tricolor* and *F. fomentarius* in our research had a higher MIC_50_ value (around 1,000 μg/mL) toward *C. acnes*. Since this bacterium was not tested in other research, it is not possible to make any comparisons. However, it should be noted that, as mentioned earlier, *D. tricolor* normally forms large numbers of relatively large fruiting bodies, and *F. fomentarius* normally forms remarkably large fruiting bodies under natural conditions. Thus, it is possible to obtain high amounts of melanin from their fruiting bodies. In addition, these two mushrooms have been widely used for medicinal purposes (Dresch et al., 2015; Wang and Zhao, 2021). Thus, the melanins from them would be safe for human applications.

In terms of biofilm eradication activity, the MBEC_50_ value of the *D. tricolor* melanin derivative (500 μg/mL) was just half of the *F. fomentarius* melanin derivative and the positive control. However, it required *D. tricolor* and *F. fomentarius* melanin derivatives at a 2-fold higher concentration (8,000 μg/mL) than that of the positive control to inhibit 90% of *C. acnes* biofilm formation. Biofilms in bacteria are responsible for two-thirds of infections, and bacterial cells in biofilms are more resistant to antibiotics by about 10 to 1,000 times (Bin et al., 2012; Haney et al., 2021). Unlike the case of evaluating antibacterial activity, methods for evaluating antibiofilm activity are not standardized. Crystal violet is one of the most commonly used dyes to stain and quantitatively analyze biofilm inhibition and eradiation. However, it is recommended that metabolic assays be run to measure the cell growth of the target bacteria to obtain a broader and more accurate picture of the activity (Haney et al., 2021). There has not been any research on the antibiofilm of melanin against *C. acnes*. We are aware of only one research investigating the antibiofilm effects of fungal melanin on some bacteria. Bin et al. (2012) reported that at a concentration of 80 μg/mL, melanin from the *A. auricula* fungus inhibited the biofilm formation of *E. coli*, *P. aeruginosa*, and *P. fluorescens* by 71.3, 61.7, and 63.2%, respectively. Notably, the melanin showed no significant inhibition of bacterial growths.

The presence of biofilm is one of the factors that facilitates the antibiotic-resistant capacity of bacteria in general and *C. acnes* in particular. Therefore, a compound that has both antibacterial activity (inhibiting the platonic cells of the pathogenic bacteria) and antibiofilm and/or biofilm eradication activity would be ideal for the treatment of those biofilm-forming bacteria. The MIC_90_ and MBEC_90_ in our research were 2-fold higher than the positive control, suggesting that it requires higher doses of them to completely kill *C. acnes* and eradicate the bacterial biofilm. However, as the MIC_50_ values of melanin derivatives of both fungal species were the same as those of the positive control and the MBEC_50_ (of the *D. tricolor* melanin derivative) was just half that of the positive control, the mechanisms of the antibacterial and antibiofilm activities of the melanin derivatives should be studied, and corresponding modifications to the samples should be made for the enhancement of their activities. In addition, the further purification of the melanin derivatives would also be an option to achieve higher activities.

### Melanins and melanin derivatives from *Daedaleopsis tricolor* and *Fomes fomentarius* are potential active ingredients for sunscreen products

Most sunscreen active ingredients prevent burning rays UVB of 290–320 nm, and far fewer have coverage of aging rays UVA of 320–400 nm (Danovaro et al., 2008). The obtained data in our research suggested that melanins from *D. tricolor* and *F. fomentarius* would have potential for broad-spectrum sunscreen products. In addition, the obtained data also revealed that melanins and melanin derivatives from these two fungi were non-toxic to skin cells.

Oh et al. (2021) reported that fungal melanin derived from *Amorphotheca resinae* has no cytotoxicity on keratinocyte HaCaT cells after 72 h of exposure, even at the highest concentration of 4 mg/mL. However, to assure the safety of using melanins and/or melanin derivatives from *F. fomentarius* and *D. tricolor* as one of the active ingredients in anti-acne sunscreens, the effects of melanins and their derivatives, as well as the blended creams containing these substances, will be tested in our follow-up research.

## Conclusion

Modification of *F. fomentarius* and *D. tricolor* melanins with arginine significantly enhanced their antibacterial and antibiofilm activities against *C. acnes* as their water solubility was improved. The antibacterial and antibiofilm activities of melanin derivatives of these two fungi were half compared with the positive control, whereas those of melanins were significantly lower. However, when it came to anti-ultraviolent activity, the melanins seemed to have better activity compared to their derivatives.

Among the two fungal species, *D. tricolor* displayed more potential. The MIC_50_ and MBEC_50_ values of the *D. tricolor* melanin derivative were 1,000 μg/mL and 250 μg/mL, respectively. In addition, at 10%, the melanin derivative of this fungal species could significantly enhance the SPF (7.74) and critical wavelength (338.67) of Nivea and turn it into a broad-spectrum sunscreen with its SPF value of 14.02 and critical wavelength of 377.0. In addition, blending the same concentration of *D. tricolor* melanin or melanin derivative also significantly increased the SPF of Olay sunscreen from 17.25 to 23.82 and 23.38, respectively.

As the melanins and melanin derivatives had negligible toxicity toward fibroblast cells (at least at the tested concentration of 100 μg/mL), they are potential candidates for anti-acne sunscreen products and worth further investigation.

## Data availability statement

The original contributions presented in the study are included in the article/Supplementary material, further inquiries can be directed to the corresponding author.

## Ethics statement

Ethical approval was not required for the studies on humans in accordance with the local legislation and institutional requirements because only commercially available established cell lines were used.

## Author contributions

TL: Data curation, Formal analysis, Methodology, Writing – original draft. NT: Data curation, Visualization, Writing – original draft. VP: Data curation, Visualization, Writing – original draft. N-DV-T: Data curation, Writing – original draft. HT: Conceptualization, Supervision, Validation, Writing – review & editing.
