# Supplementary material for: Anti-ultraviolet, antibacterial, and biofilm eradication activities against Cutibacterium acnes of melanins and melanin derivatives from Daedaleopsis tricolor and Fomes fomentarius
Source: Front Microbiol. 2024 Jan 8;14:1305778. doi: 10.3389/fmicb.2023.1305778 (PMC10803019; doi:10.3389/fmicb.2023.1305778)
Supplement: Supplementary file 1 [file Data_Sheet_1.PDF]

Identification of the fungal samples used in the research

CH2 (*Daedaleopsis tricolor*)

ITS sequence

TGTAGCTGGCCTTCCGAGGCATGTGCACGCCCTGCTCATTCCACTCTACACCTGTGC
ACTTACTGTGGGTCTCAGGCGAGCGTCGGTCGCTTCGCGGCGTCGTCGTTCAACTGG
GCTCACGTTTTACTACAAACTATTAAAGTATCAGAATGTCTACTGCGAATTAACGCA
TTTAAATACAACTTTCAGCAACGGATCTCTTGGCTCTCGCATCGATGAAGAACGCAG
CGAAATGCGATAAGTAATGTGAATTGCAGAATTCAGTGAATCATCGAATCTTTGAAC
GCACCTTGCGCTCCTTGGTATTCCGAGGAGCATGCCTGTTTGAGTGTCATGAAATTCT
CAACCTAACGAGTCTTTGCGGGCTCGGTAGGCTTGGACTTGGAGGTTCTTGTCGGCC
TAACGGTCGGCTCCTCTTAAATGCATTAGCTCGGTTCCTTGCGGATCGGCTCACGGT
GTGATAATTGTCTACGCCGCGACCGTTGAAGCGTTTTGGCCGGCTTCTAATCGTCTC
GTTGGAGACACTACTCTTTATGACCTTCTGACCTCAAATCAGGTAGGACTACCCGCT
GAACTTAAGCATATC

Image of fruiting bodies

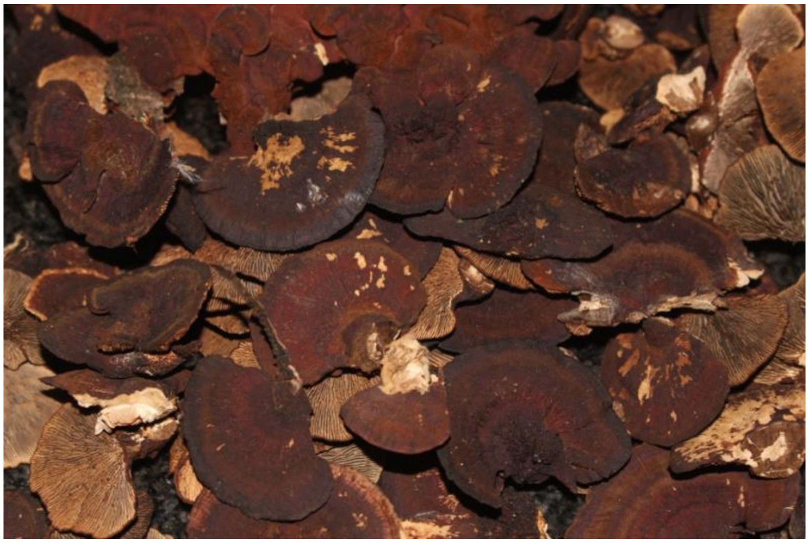

CH3 (*Fomes fomentarius*)

ITS sequence

TTGTAGCTGGCCTTCCGAGGCATGTGCACGCCCTGCTCATCCACTCTACACCTGTGC
ACTTACTGTGGGATTTCAGGTGCGTCGCTTTGCGGCGGCGTCACTCGGCCCACGTTTT
CTTTACAAACTATTGAAGTAACAGAATGTTTATTGATGTAACGCATCTATAATACAA
CTTTCAGCAACGGATCTCTTGGCTCTCGCATCGATGAAGAACGCAGCGAAATGCGAT
AAGTAATGTGAATTGCAGAATTCAGTGAATCATCGAATCTTTGAACGCACCTTGCGC
TCCTTGGTATTCCGAGGAGCATGCCTGTTTGAGTGTCATGAAATTCTCAACCTATAA
ACTTTTGCGGGTTTGTAGGGTTGGCTATTGGAGGCTTTTGCTGGCCCTCGTTTGAGTC
AGCTCCTCTCAAATGCATTAGCTTGGTTCCTTGCGGATCGGCTGTCGGTGTGATAAT
GTCTACGCCGCGACCGTGAAGCGTTTGGAGAGCTTCTAATGGTCTCGTCAGAGACAG
CTTTTATGAACTCTGACCTCAAATCAGGTAGGACTACCCGCTGAACTTAAGCATATC
AA

Image of fruiting body

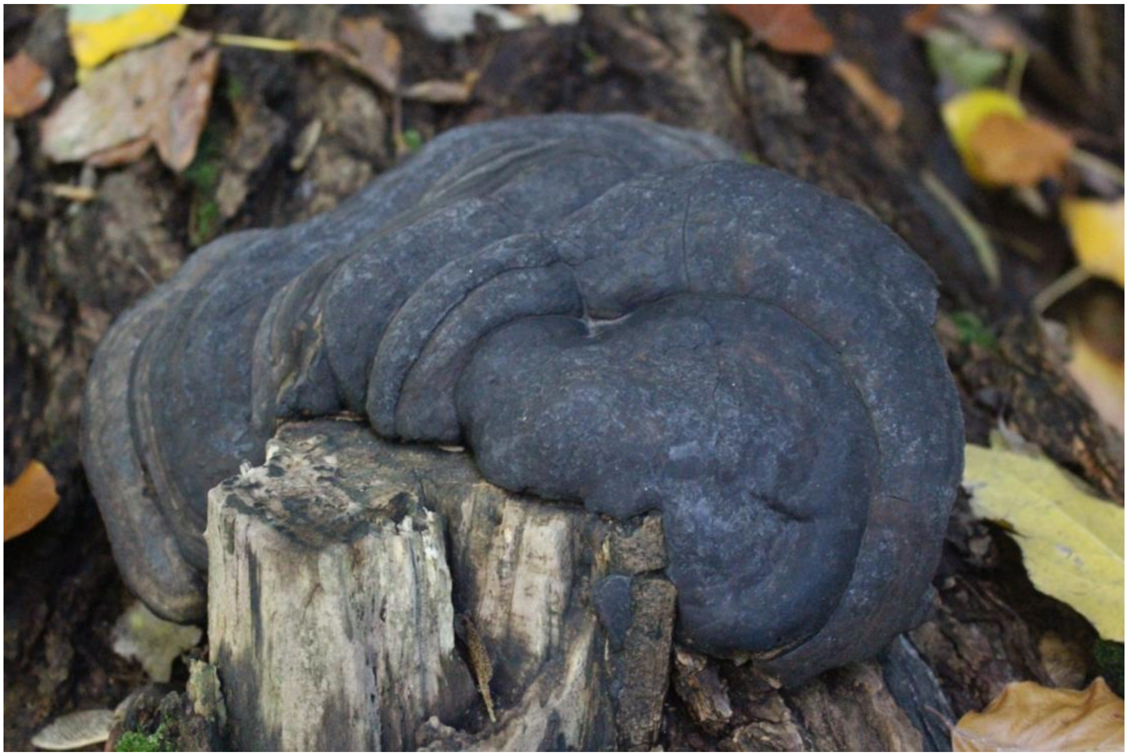

BLAST results on NCBI.

Sample	Name	Query cover	Per. ident	Accession
**CH2**	*Daedaleopsis tricolor* isolate DT2017230	100%	100%	MN636257
	Daedaleopsis tricolor isolate DT2017255	100%	100%	MN636256
	Daedaleopsis confragosa isolate DC2017186	100%	100%	MN636248
**CH3**	*Fomes fomentarius* isolate SP3	100%	100%	MN065451
	Fomes fomentarius voucher Cui 8020	100%	99.48%	JX290073
	Fomes fomentarius strain LE-BIN 4762	100%	99.83%	OL764369
