# Supplementary material for: Anti-ultraviolet, antibacterial, and biofilm eradication activities against Cutibacterium acnes of melanins and melanin derivatives from Daedaleopsis tricolor and Fomes fomentarius
Source: Front Microbiol. 2024 Jan 8;14:1305778. doi: 10.3389/fmicb.2023.1305778 (PMC10803019; doi:10.3389/fmicb.2023.1305778)
Supplement: Supplementary file 2 [file Table_1.DOCX]

**Table S1.** Ingredients of the melanin/derivative blended creams

| **Ingredient** | 4% blended cream | 7% blended cream | 10% blended cream |
| --- | --- | --- | --- |
| Melanin or melanin derivative (g) | 0.001 | 0.00175 | 0.0025 |
| 0.1M NaOH or distilled water (mL) | 1 | 1 | 1 |
| Desired concentration of melanin suspension (g/mL) | 0.001 | 0.00175 | 0.0025 |
| Cream base (g) | 0.025 | 0.025 | 0.025 |
