# Supplementary material for: Anti-ultraviolet, antibacterial, and biofilm eradication activities against Cutibacterium acnes of melanins and melanin derivatives from Daedaleopsis tricolor and Fomes fomentarius
Source: Front Microbiol. 2024 Jan 8;14:1305778. doi: 10.3389/fmicb.2023.1305778 (PMC10803019; doi:10.3389/fmicb.2023.1305778)
Supplement: Supplementary file 3 [file Table_2.DOCX]

| **Arginine concentration (µg/mL)** | **OD_sample_ / OD_negative control_** |
| --- | --- |
| 62.5 | 1.0438 ± 0.0488 |
| 125 | 0.9921 ± 0.0301 |
| 250 | 1.0332 ± 0.0634 |
| 500 | 1.0594 ± 0.0547 |
| 1000 | 1.0454 ± 0.0868 |
| 2000 | 1.0224 ± 0.0466 |
| 4000 | 0.9994 ± 0.0292 |

**Table S2.** The insignificant difference in the growth of C. acnes with and without arginine addition.
