# Supplementary material for: Anti-ultraviolet, antibacterial, and biofilm eradication activities against Cutibacterium acnes of melanins and melanin derivatives from Daedaleopsis tricolor and Fomes fomentarius
Source: Front Microbiol. 2024 Jan 8;14:1305778. doi: 10.3389/fmicb.2023.1305778 (PMC10803019; doi:10.3389/fmicb.2023.1305778)
Supplement: Supplementary file 4 [file Table_3.DOCX]

**Table S3.** The insignificant difference in biofilm formation of C. acnes with and without arginine addition.

| **Arginine concentration (µg/mL)** | **OD_sample_ / OD_negative control_** |
| --- | --- |
| 31.25 | 1.0228 ± 0.0346 |
| 62.5 | 1.0067 ± 0.0226 |
| 125 | 1.0081 ± 0.0415 |
| 250 | 0.9954 ± 0.0459 |
| 500 | 0.9988 ± 0.0284 |
| 1000 | 1.0313 ± 0.0199 |
| 2000 | 1.0024 ± 0.0261 |
| 4000 | 0.9906 ± 0.0112 |
| 8000 | 0.9842 ± 0.0188 |

Note: The sample consisted of distilled water with arginine; the negative control was distilled water alone.

No significant differences were seen in all concentrations of L-arginine (p = 0.634 > 0.05). All the obtained ratios were observed to approximately be equal to 1, indicating that the biofilm growths in wells containing arginine and negative control were almost similar; in other words, the biofilm formation of *C. acnes* was not affected by L-arginine.
